# Comparison between MALDI-TOF MS and MicroScan in the identification of emerging and multidrug resistant yeasts in a fourth-level hospital in Bogotá, Colombia

**DOI:** 10.1186/s12866-019-1482-y

**Published:** 2019-05-23

**Authors:** Andrés Ceballos-Garzón, Gloria Cortes, Florent Morio, Edna L. Zamora-Cruz, Melva Y. Linares, Beatriz E. Ariza, Sandra L. Valderrama, Javier R. Garzón, Carlos A. Alvarez-Moreno, Patrice Le Pape, Claudia M. Parra-Giraldo

**Affiliations:** 1 Departamento de Microbiología, Facultad de Ciencias, Unidad de Proteomica y Micosis Humanas, Grupo de Enfermedades Infecciosas, Pontificia Universidad Javeriana, Bogotá, Colombia; 2 Laboratorio Clínico, Área de Microbiología, Hospital Universitario San Ignacio, Bogotá, Colombia; 3 Unidad de Infectología, Departamento de Medicina Interna, Facultad de Medicina, Hospital Universitario San Ignacio, Bogotá, Colombia; 4 Grupo de Investigación en Enfermedades Infecciosas, Hospital Universitario San Ignacio, Bogotá, Colombia; 5 Department of Parasitology and Medical Mycology of the University of Nantes, Nantes Atlantique Universities, Faculty of Pharmacy, Nantes, France

**Keywords:** Comparison, MALDI-TOF MS, MicroScan, Unusual yeast, Yeast identification

## Abstract

**Background:**

The introduction of MALDI-TOF MS in the clinical microbiology laboratory has modified the approaches for the identification of fungi. Thanks to this tool, it is possible to identify cryptic species, which possess critical susceptibility patterns. Clinical strains were identified using the MicroScan and MALDI-TOF MS systems. Discrepant results from both methods were investigated using ITS rDNA barcoding. Finally, these isolates were also tested for in vitro susceptibility.

**Results:**

The percentage of agreement between both methods to 498 yeast isolates was of 93.6% (32 discrepant isolates). The concordance of ITS sequencing with MALDI-TOF MS was higher (99%) than that of MicroScan (94%). Several of these discordant yeasts displayed high MICs for antifungal agents.

**Conclusions:**

Our study highlights the need of the MS and molecular approaches such as MALDI-TOF MS and ITS rDNA barcoding for the correct identification of emerging or cryptic yeast species; besides, some of these could be multidrug resistant.

This work was the first experience in the implementation of the MALDI-TOF MS technology in Colombia. We found the first uncommon yeasts including *Candida auris* and we could identify *Trichosporon faecalis*. Our work highlights a clear necessity of an accurate yeast identification as a much more pertinent technique than the susceptibility profiles, because the most unusual yeasts exhibit resistance profiles to the few available antifungals.

## Background

In the last decades, the incidence of invasive fungal infections has progressively increased, especially in critically ill and immunocompromised patients [1, 2]. Yeast infections are mostly caused by *Candida* followed by *Cryptococcus*, and less frequently by *Rhodotorula*, *Saccharomyces*, *Trichosporon* and *Pichia*. These emerging species are also contributing to the epidemiological changes recorded in recent years, which significantly impact therapeutic regimens in patients as some yeasts can exhibit innate drug resistance [3–6].

Within the genus *Candida*, *C. albicans* is still the most common fungal pathogen worldwide. Despite being part of the mucocutaneous, gastrointestinal and genitourinary mycobiota in humans, *C. albicans* can be responsible for nearly 50% of candidaemia. In the 90’s the prevalence of candidaemia was between 64 and 48% but this percentage decreased to 38% during the period 2008–2011 due to increased prevalence of non *C. albicans* species [5, 7, 8]. For *Cryptococcus,* reports indicate a prevalence of 4%. Such a low percentage has not ever been described for emerging species [9–11].

The accurate identification of the fungal species that cause the infection is of paramount importance since the necessity to initiate the appropriate antifungal therapy. Furthermore, the time required for diagnosis/identification is also critical as any delay will affect the prognosis dramatically when dealing with invasive candidiasis as well as complicate a relevant stewardship [12, 13].

On South America mycological identification usually relies on phenotypic, biochemical, enzymatic and immunological approaches [14]. The MicroScan system is a rapid technology for microbial identification able to provide results after 4 h of inoculation [15]. Only available for yeast identification, this method relies on enzymatic reactions in a panel. The enzyme activities of each isolate are determined by colour changes in the chromogenic substrates (or a pH indicator). The biochemical reactions generate numerical profiles, which are then compared with a numerical database. In recent years, Mass Spectrometry (MS) using MALDI-TOF MS technology has been increasingly used as a tool for microbiological identification due to its high performance and less time required when compared with conventional methods [16].

Our aim was to compare the performance of MicroScan with that of MALDI-TOF MS for yeast identification. This study was conducted on a large collection of clinical isolates collected prospectively at the San Ignacio Hospital, Bogota, Colombia. Those isolates yielding discrepant results were further identified by the gold standard ITS and D1-D2 rDNA barcoding. Because of the few data on rare/emerging yeasts, susceptibility profiles were also determined.

## Results

### Performance of MicroScan in comparison with Bruker MALDI-TOF MS

With the MicroScan technology 497/498 (99.7%) strains were identified, belonging to 16 distinct species from 7 genera. When performing MALDI-TOF MS identification with the Bruker instrument 494 /498 (99%) strains were identified, belonging to 21 distinct species from 6 genera. The percentage of agreement between both methods was 93.5% (466 isolates) (Table 1). The remaining 32 isolates yielding discrepant results (*n* = 27) or being no-identified by one of the two methods were subjected to further investigations for molecular identification as the gold standard.

**Table 1:** Matrix of concordance identification from MicroScan technology against MALDI-TOF MS technology

Genus/Species	MALDI-TOF-MS (BRUKER BIOTYPER)																						Overall MicroScan	Discrepancies or no-identification
	*Candida albicans*	*Candida auris*	*Candida dubliniensis*	*Candida glabrata*	*Candida guilliermondii*	*Candida intermedia*	*Candida kefyr*	*Candida krusei*	*Candida lusitaniae*	*Candida metapsilosis*	*Candida nivariensis*	*Candida orthopsilosis*	*Candida parapsilosis*	*Candida tropicalis*	*Cryptococcus gattii*	*Cryptococcus neoformans*	*Cryptococcus neoformans* var. *grubii*	*Geotrichum candidum*	*Pichia kluyveri*	*Rhodotorula mucilaginosa*	*Saccharomyces cerevisiae*	Unidentified		
MicroScan (Beckman Coulter)																								
*Blastoschizomyces* spp*.*																						1	1	1
*Candida albicans*	305		4																				309	4
*Candida catenulata*		2																					2	2
*Candida famata*		1				4																	5	5
*Candida glabrata*				40							1												41	1
*Candida guilliermondii*					6																		6	0
*Candida inconspicua*		1																	1				2	2
*Candida kefyr*							3																3	0
*Candida krusei*								8															8	0
*Candida lusitaniae*									5														5	0
*Candida parapsilosis*										4		2	26										32	6
*Candida tropicalis*														53									53	0
*Cryptococcus neoformans*															2	9	2						13	4
*Geotrichum* spp.																		2					2	2
*Rhodotorula rubra*																				1			1	1
*Saccharomyces cerevisiae*																					11		11	0
*Trichosporon beigelii*																						3	3	3
Unidentified		1																					1	1
Overall MALDI-TOF MS	305	5	4	40	6	4	3	8	5	4	1	2	26	53	2	9	2	2	1	1	11	4	**498**	**32**

### Resolution of the discrepancies by sequencing

The 32 discordant identifications occurred mostly with rare and/or emergent species. Table 2, 3 presents the total and individual cases of discordance, and the species with which there was confusion**.** The vast majority of errors (*n* = 30) occurred with the MicroScan system while MALDI-TOF MS Bruker showed the highest agreement with ITS rDNA barcoding. Using the Bruker Biotyper instrument, the four isolates (0.8%) yielding “no identification” (score < 1.7) were finally identified by sequencing as *Saprocheta suaveolens* (*n* = 1) and *Trichosporon faecale* (*n* = 3). Notably, the MALDI-TOF MS Bruker could identify several cryptic species such as the multidrug resistant *C. auris* (*n*  =  5), *C. intermedia* (*n*  =  3), *C. metapsilosis* (*n*  =  4), *C. nivariensis* (*n*  =  1), *C. orthopsilosis* (*n*  =  2), *C. gattii* (*n*  =  2), *C. neoformans* var. *grubii* (*n*  =  2*),* and *G. candidum* (*n* = 2) (Tables 2, 3).

**Table 2:** Comparative analysis of yeast identification by MALDI-TOF MS and MicroScan systems

Genus/Species	Total	MicroScan (Beckman Coulter)				MALDI-TOF MS (Bruker)			
		Agreement	False	No ID	Total	Agreement	False	No ID	Total
*Blastoschizomyces* spp.									
*Blastoschizomyces capitatus*	0	0	1	0	1	0	0	0	0
*Candida* spp.									
*Candida albicans*	305	305	4	0	309	305	0	0	305
*Candida auris*	5	0	0	1	5	5	0	0	5
*Candida dubliniensis*	4	0	0	0	0	4	0	0	4
*Candida catenulata*	0	0	2	0	2	0	0	0	0
*Candida famata*	0	0	5	0	5	0	0	0	0
*Candida glabrata*	40	40	1	0	41	40	0	0	40
*Candida guilliermondii*	6	6	0	0	6	6	0	0	6
*Candida inconspicua*	0	0	2	0	2	0	0	0	0
*Candida intermedia*	3	0	0	0	0	3	1	0	4
*Candida kefyr*	3	3	0	0	3	3	0	0	3
*Candida krusei*	8	8	0	0	8	8	0	0	8
*Candida lusitaniae*	5	5	0	0	5	5	0	0	5
*Candida metapsilosis*	5	0	0	0	0	4	0	0	4
*Candida nivariensis*	1	0	0	0	0	1	0	0	1
*Candida orthopsilosis*	2	0	0	0	0	2	0	0	2
*Candida parapsilosis*	26	26	6	0	32	26	0	0	26
*Candida tropicalis*	53	53	0	0	53	53	0	0	53
*Cryptococcus* spp.									
*Cryptococcus gattii*	2	0	0	0	0	2	0	0	2
*Cryptococcus neoformans*	9	11	2	0	13	9	0	0	9
*Cryptococcus neoformans* var. *grubii*	2	0	0	0	0	2	0	0	2
*Geotrichum* spp.									
*Geotrichum* sp*.*	0	0	2	0	2	0	0	0	0
*Geotrichum candidum*	2	0	0	0	0	2	0	0	2
*Pichia* spp.									
*Pichia kluyveri*	1	0	0	0	0	1	0	0	1
*Rhodotorula* spp.									
*Rhodotorula rubra*	0	0	1	0	1	0	0	0	0
*Rhodotorula mucilaginosa*	1	0	0	0	0	1	0	0	1
*Saccharomyces* spp.									
*Saccharomyces cerevisiae*	11	11	0	0	11	11	0	0	11
*Saprochaete* spp.									
*Saprochaete suaveolens*	1	0	0	0	0	0	0	1	1
*Trichosporon* spp.									
*Trichosporon faecale*	3	0	0	0	0	0	0	3	3
*Trichosporon beigelii*	0	0	3	0	3	0	0	0	0
Overall (%)	498 (100)	468 (94)	29 (5.8)	1 (0.2)	498 (100)	493 (99)	1 (0.2)	4 (0.8)	498 (100)

**Table 3:** Molecular biology results resolve discrepencies or no- identifications by MicroScan and MALDI-TOF MS

ID Molecular biology Sequencing 16S	Micro Scan	MALDI-TOF MS
*Candida auris* (5)	*Candida catenulata* (2) *Candida famata* (1) *Candida Incospicua* (1) Unidentified (1)	*Candida auris* (5)
*Candida intermedia* (3)	*Candida famata* (3)	*Candida intermedia* (3)
*Candida dubliniensis* (4)	*Candida albicans* (4) *Cryptococcus laurenti* (1)	*Candida dubliniensis* (4)
*Candida nivariensis* (1)	*Candida glabrata* (1)	*Candida nivariensis* (1)
*Candida metapsilosis* (5)	*Candida parapsilosis* (4) *Candida famata* (1)	*Candida metapsilosis* (4) *Candida intermedia* (1)
*Candida orthopsilosis* (2)	*Candida parapsilosis* (2)	*candida orthopsilosis* (2)
*Crytococcus neoformans* (2)	*Cryptococcus neoformans* (2)	*Cryptococcus neoformans* var. *grubii* (2)
*Cryptococcus gatti* (2)	*Cryptococcus neoformans* (2)	*Cryptococcus gatti* (2*)*
*Geotrichum candidum* (2)	*Geotrichum* sp. *(*2)	*Geotrichum candidum* (2)
*Pichia kluyveri* (1)	*Candida incospicua* (1)	*Pichia kluyveri* (1)
*Rhodotorula mucilaginosa* (2)	*Rhodotorula rubra* (1)	*Rhodotorula mucilaginosa* (1)
*Trichosporon faecale* (3)	*Trichosporon beigelii* (3)	unidentified (3)
*Saprochaete suaveolens* (1)	*Blastoschizomyces capitatus* (1)	unidentified (1)

### In vitro antifungal susceptibility

We assessed the susceptibility profiles of isolates that showed discrepancies in their identification. Among *Candida* yeasts, *C. auris* presented the highest MIC values for FLU and AMB; *C. metapatilosis* for AMB and ITC. *C. dubliniensis* was the only species with a low MIC for VRC. *C. intermedia* and *C. nivariensis* were the only two species susceptible to all of the drugs evaluated (Table 4).

**Table 4:** Susceptibility profiles of discrepant *Candida* isolates

Molecular identification *(n=)*	*Strain*	Antifungal drugs tested					
		FLU	ITC	VRC	AMB	CAS	AFG
*Candida auris (5)*	*1*	24	0.25	0.64	0.75	0.47	0.012
	*2*	12	0.25	0.064	1.5	0.094	0.19
	*3*	> 256	0.25	0.094	0.75	0.032	0.008
	*4*	12	0.38	0.047	0.75	0.094	0.004
	*5*	> 256	0.25	0.047	0.75	0.032	0.047
*Candida dubliniensis (4)*	*1*	0.19	0.092	0.004	0.16	0.012	0.006
	*2*	0.19	0.016	0.003	0.064	0.023	0.002
	*3*	0.19	0.032	0.003	0.094	0.023	0.002
	*4*	0.25	0.047	0.004	0.125	0.25	0.003
*Candida intermedia (3)*	*1*	0.125	0.047	0.012	0.064	0.047	0.003
	*2*	1.5	0.125	0.012	0.094	0.064	0.006
	*3*	3	0.032	0.064	0.25	0.19	0.023
*Candida metapsilosis (5)*	*1*	3	0.094	0.047	2	0.75	0.19
	*2*	3	0.125	0.094	0.38	0.19	0.23
	*3*	6	0.25	0.094	0.002	0.125	0.047
	*4*	0.75	0.094	0.25	0.75	0.25	0.19
	*5*	2	0.047	0.064	0.032	0.19	0.032
*Candida nivariensis (1)*	*1*	0.75	0.125	0.023	0.50	0.047	0.004
*Candida orthopsilosis (2)*	*1*	4	0.38	0.125	0.75	1.5	1
	*2*	4	0.38	0.064	0.5	0.064	0.38

*FLU* fluconazole, *ITC* itraconazole, *VRC* voriconazole, *AMB* amphotericin B, *CAS* caspofungin, *AFG* anidulafungin

On the other hand, all the other yeasts (non *Candida*) showed higher MIC values for FLU and generally for azoles but were sensitive to AMB. All isolates had high MICs for echinocandins except *P. kluyveri* (Table 5).

**Table 5:** Susceptibility profiles of discrepant non-*Candida* isolates

Molecular Identification *(n=)*	*Strain*	Antifungal drugs tested					
		FLU	ITC	VRC	AMB	CAS	AFG
*Trichosporon faecalis (3)*	*1*	8	1.25	0.094	0.50	> 32	> 32
	*2*	16	1	0.125	0.75	> 32	> 32
	*3*	12	1	0.094	1	> 32	> 32
*Cryptococcus gattii (2)*	*1*	12	0.19	0.064	0.19	> 32	> 32
	*2*	32	0.25	0.36	0.094	> 32	> 32
*Cryptococcus neoformans (2)*	*1*	6	0.094	0.032	0.002	> 32	> 32
	*2*	6	0.064	0.047	0.19	> 32	> 32
*Saprochaete suaveolens (1)*	*1*	> 32	0.032	0.125	0.25	> 32	> 32
*Geotrichum candidum (2)*	*1*	> 256	0.25	0.047	0.125	0.38	0.016
	*2*	4	1.5	0.25	1	> 32	> 32
*Rhodotorula mucilaginosa (1)*	*1*	> 256	> 32	> 32	0.125	> 32	> 32
*Pichia kluyveri (1)*	*1*	> 256	> 32	> 32	0.064	0.064	0.004

*FLU* fluconazole, *ITC* itraconazole, *VRC* voriconazole, *AMB* amphotericin B, *CAS* caspofungin, *AFG* anidulafungin

## Discussion

Proteomic analysis using the MALDI-TOF MS methodology offers a great opportunity for identifying microorganisms that are difficult to identify by biochemical methods. In this study, we compared two methodologies for the identification of clinically relevant yeasts: the Microscan system (biochemical approach) and the Bruker MS system (proteomic approach). This study also confirmed the wide diversity of species obtained from patients in the clinical setting, highlighting the importance of a correct identification at the species level for the determination of the appropriate therapy (since cryptic species may have non-predicted susceptibility profiles). The correct identification of clinical yeast isolates has become essential for optimal clinical management, as well as for detailed epidemiological studies and the prevention and containment of outbreaks. It is in our interest to analyse in depth the cases where the identification errors are important in the treatment, as it is in the case of the isolations of *C. auris*, as a misidentification yeast by conventional technology [17].

A correct identification by morphological and conventional testing with MicroScan was achieved for 94% of the isolates, a result similar (96% of *n* = 357 clinical isolates) to a previously reported one by other authors using the same system [15]. The automated system MicroScan handles 10% of colorimetric biochemistry and 90% of enzymatic biochemistry; the latter are reversible tests depending on the reading time. This is of great importance since if there is no identification in 4 h (time indicated by the commercial house) the biochemical reactions can revert themselves, leading to false negative results. In addition, the performances of this phenotypic system are intimately dependent on regular updates of the database. Since MicroScan is not capable of identifying *Geotrichum* spp. (at the species level), we recorded those isolate identifications as erroneous. In addition, cryptic or recently emerging species such as *C. auris*, *C. intermedia C. metapsilosis*, *C. nivariensis, C. orthopsilosis*, *C. gattii* and *S. suaveolens*, which are not yet present in the MicroScan database version 4.11.1020.1 cannot be readily identified.

MALDI-TOF MS systems have recently been developed and implemented in diagnostic microbiology laboratories for bacterial and fungal identification due to their efficacy, rapidity and minimum hands-on time. This technology, as highlighted here, provides valid results for most yeast species (84–99%). However, optimal results depend on the robustness of the system libraries [18, 19]. For those reasons, the accuracy of the MALDI-TOF MS System is modifying the way in which yeast identification is being performed, surpassing the conventional techniques. In this study, MALDI-TOF MS could correctly identify 98.9 of the strains analysed. The 5 errors were due to a misidentification of *C. metapsilosis* and the lack of identification of *Trichosporon faecale* and *Saprochaete suaveolens* even though they are present in the Biotyper database. We believe that the error was possibly due to the frequent difficulty in protein extraction for these species. In the case of *Saprochaete suaveolens* the database is not robust thus leading to erroneous results. A solution to the identification of these microorganisms is the creation or improvement of libraries [20].

Regarding susceptibility the change in the epidemiology of *Candida* species has occurred at the same time with the emergence of *Candida* strains resistant to antifungal drugs mainly to fluconazole, which is used as the first line pharmacological treatment [21, 22]. In this study, we did not handle cut-off points in the interpretation of susceptibility profiles since some of the yeasts do not have an established MIC [23]. It is important to mention that in all cases the non-albicans yeasts showed high MIC values, at least for one of the antifungals evaluated.

Successful treatment of fungal infections depends on the early identification of species and patterns of sensitivity to antifungal agents. The growth rate of resistance confirms the importance of monitoring changes in the distribution of pathogenic species. The sensitivity pattern of the *Candida* species revealed in this study shows that amphotericin B, voriconazole and caspofungin with the lowest MICs appear to be suitable drugs. Moreover, as expected from previous studies, for most of non-Candida species, amphotericin B was the antifungal displaying the highest in vitro activity.

In conclusion, yeast identification has advanced enabling the recognition of new “cryptic species”. These yeasts are yet not easily identifiable by traditional phenotypic methods commonly employed in clinical laboratories. In this study, we have shown the high performance of MALDI-TOF MS technology for the identification of clinically relevant yeasts. Moreover, continued increase on the number of susceptible patients and the selection pressures imposed using antifungal drugs continue to result in the emergence of new microorganisms that cannot be easily identified in laboratories. It is important that physicians take into account the identification of pathogenic yeasts down to the species level due to the diversity of antifungal sensitivity profiles.

## Conclusions

Our study highlights the need of the MS and molecular approaches such as MALDI-TOF MS and ITS rDNA barcoding for the correct identification of emerging or cryptic yeast species; besides, some of these could be multidrug resistant.

## Methods

### Study design

The study was a single-center prospective analysis covering the period from November of 2013 to March of 2015. All the yeast isolates obtained from hospitalized immunocompromised patients of the San Ignacio Hospital were included in the present study. Yeasts were identified at the Laboratory of Microbiology of the San Ignacio Hospital using MicroScan (MicroScan WalkAway-96 Plus, Siemens, Deerfield, IL, USA) and at the Laboratory of Proteomics and Human Mycoses of the Faculty of Science of the Pontificia Universidad Javeriana using MALDI-TOF MS (Bruker Daltonics, Bremen, Germany).

A total of 498 clinical isolates were recovered from different samples such as respiratory secretions (*n* = 168), stool (*n* = 133), urine (*n* = 55), blood (*n* = 50), swabs (*n* = 44), biopsies (*n* = 24), medical devices (*n* = 10), cerebrospinal fluids (*n* = 10), abdominal fluids (*n* = 3) and bone marrow (*n* = 1). Species identification was performed using MicroScan in parallel with MALDI-TOF MS. Discrepant isolates were subjected to molecular identification as the gold standard.

### MicroScan WalkAway system

The MicroScan system for yeast identification consists of a 96-well microdilution plate with 27 dehydrated chromogenic substrates. The enzyme activity of each isolate is determined by colour change. Isolates were previously grown on Sabouraud dextrose agar (SDA) at 30 °C for 24–36 h. Suspensions were prepared and calibrated against the MicroScan turbidity standard, and the panel was incubated aerobically for 4 h at 37 °C. Biochemical reactions generate numerical profiles that are compared with a database to identify organisms [15]. Urease assimilation and growth on canavanine-glycine-bromothymol blue agar confirmed the identification for *Cryptococcus* isolates.

## MALDI-TOF MS

### Bruker Biotyper

After incubation of clinical strains at 35 °C for 24–36 h on SDA, protein extraction was performed using the formic acid/ethanol method according to the Bruker Daltonics protocol. Briefly, two or three colonies were mixed with 300 μl of HPLC grade water until homogenization and then 600 μl of 100% ethanol (Sigma-Aldrich, St Louis, MO) were added. After centrifugation at 15,000 g for 2 min, the pellet was dried at 25 °C, reconstituted in equal volumes of 100% formic acid (Sigma- Aldrich) and acetonitrile (Sigma-Aldrich) (20 μL each), mixed thoroughly, and centrifuged at 15, 000 g for 2 min. One microliter of supernatant was spotted onto a 96-spot steel plate (Bruker Daltonik) and allowed to dry at room temperature before the addition of 1 mL of the HCCA matrix (provided by the supplier). Each sample was tested in duplicate. Only the spot returning the highest probability score of identification was considered [24]. The protein mass spectra was analysed using the Flex Control software and the MALDI Biotyper version 3.1 7311 reference spectra (main spectra) (Bruker Daltonics, Bremen, Germany). MALDI-TOF MS results were then compared and a score was obtained according to the manufacturer’s technical specifications, as follows: correct genus and species identification (≥2.0), correct genus identification (1.7–2.0), and no reliable identification (< 1.7) [25]. The 498 clinical isolates was a score above 2.

### Molecular identification

Molecular identification of discrepant isolate results between both methods was subjected to molecular identification by amplification and sequencing of the ITS rDNA regions without prior DNA extraction step (colony PCR) [26]. Amplification of the ITS rDNA was achieved using the universal primers ITS1 (TCCGTAGGTGAACCTGCGG) and ITS4 (TCCTCCGCTTATTGATATGC) [27]. Nucleotide sequences were assembled using the Seq Scape software (Applied Biosystems, Foster City, CA, USA.) and compared with the GenBank database using the BLAST algorithm or with the MycoBank database. For some isolates, identification was further confirmed by amplification of the D1-D2 region of the 28S rDNA using the primers NL1 (GCATATCAATAAGCGGAGGAAAAG) and NL4 (GGTCCGTGTTTCAAGACGG) [27]. A similarity ≥98% between the unknown sequence and the closest matching sequence from the reference database was used as the criterion to identify an isolate to the species level. Additional primer pairs targeting the IGS1, namely IGS1-F (ATCCTTTGCAGACGACTTGA) and IGS1-R (GTGATCAGTGCATTGCATGA) for *Cryptococcus*, and 26SF (ATCCTTTGCAGACGACTTGA) and (AGCTTGACTTCGCAGATCGG) for *Trichosporon* were used to obtain a reliable identification at the species level [28, 29].

### In vitro antifungal susceptibility testing

Each isolate displaying discrepant results was subjected to in vitro susceptibility testing using the E-test (Biomérieux) against triazoles (fluconazole, itraconazole and voriconazole), echinocandins (anidulafungin and caspofungin), and amphotericin B, according to the manufacturer’s instructions. The MIC was read as the drug concentration that leads to complete inhibition (100%) for amphotericin B, and 80% inhibition for azoles and echinocandins. Two control strains (*C. parapsilosis* ATCC 22019 and *C. krusei* ATCC 6258) were included in each set of experiments.

## Abbreviations

AMB: Amphotericin B
FLU: Fluconazole
ITC: Itraconazole
ITS: Internal transcribed spacer
MALDI-TOF MS: Matrix-assisted laser desorption/ionization time-of-flight mass spectrometry
MIC: Minimum inhibitory concentration
VRC: Voriconazole
